# A central nervous system-focused treatment approach for people with frozen shoulder: protocol for a randomized clinical trial

**DOI:** 10.1186/s13063-019-3585-z

**Published:** 2019-08-13

**Authors:** Enrique Lluch-Girbés, Lirios Dueñas, Silvia Mena-del Horno, Alejandro Luque-Suarez, Santiago Navarro-Ledesma, Adriaan Louw

**Affiliations:** 1 Department of Physiotherapy, University of Valencia, Valencia, Spain; 2 Department of Physiotherapy, Human Physiology and Anatomy, Faculty of Physical Education & Physiotherapy, Vrije Universiteit Brussel, Brussels, Belgium; 3 Pain in Motion International Research Group, http://www.paininmotion.be; 4 Physiotherapy in Motion, Multi speciality Research Group (PTinMOTION), Malaga, Spain; 5 Department of Physiotherapy, Universidad de Malaga, Andalucia TECH, Malaga, Spain; 6 Instituto de la Investigacion Biomedica de Malaga (IBIMA), Malaga, Spain; 7 Department of Physiotherapy, University of Granada, Malaga, Spain; 8 International Spine and Pain Institute, Story City, Iowa USA

**Keywords:** Shoulder pain, Shoulder adhesive capsulitis, Central nervous system, Physiotherapy

## Abstract

**Background:**

Frozen shoulder (FS) is a musculoskeletal condition of poorly understood etiology that results in shoulder pain and large mobility deficits. Despite some physical therapy interventions, such as joint mobilization and exercise, having shown therapeutic benefit, a definitive treatment does not currently exist. The aim of this study will be to compare the effectiveness of a central nervous system (CNS)-directed treatment program versus a standard medical and physical therapy care program on outcomes in participants with FS.

**Methods/design:**

The study is a two-group, randomized clinical trial with blinding of participants and assessors. Participants will be recruited via referrals from orthopedic surgeons and physical therapists, community-based advertisements, private care practices and hospitals. Participants will be randomized to receive either a CNS-focused treatment program or standard medical and physical therapy care. The Shoulder Pain And Disability Index (SPADI) will be the primary outcome, while the Numeric Pain Rating Scale (NPRS), shoulder range of movement (ROM), The Patient Specific Functional Scale, two-point discrimination threshold and laterality judgement accuracy will be the secondary outcomes. Assessment will occur at baseline, at the end of the treatment program (week 10), and at 3 and 6 months’ follow-up.

**Discussion:**

Preliminary data suggest that treatments that target CNS function are a promising approach to the treatment of people with shoulder pain including patients with FS. In the context of modest effects from most available physical therapy treatments for FS, this CNS-focused approach may lead to improved clinical outcomes. The trial should determine if the CNS-directed program is more effective than traditional interventions at reducing pain intensity and improving function in a FS cohort and will follow up participants for 6 months, providing important information on the persistence of any treatment effects.

**Trial registration:**

NCT03320200. Registered on October 25, 2017.

**Electronic supplementary material:**

The online version of this article (10.1186/s13063-019-3585-z) contains supplementary material, which is available to authorized users.

## Key points

- The effects of central nervous system (CNS) treatment on frozen shoulder will be analyzed
- Graded sensory discrimination and Graded Motor Imagery trainings will be applied
- Outcome measures will be shoulder pain and disability

## Background

Frozen shoulder (FS) is a musculoskeletal condition of poorly understood etiology that results in shoulder pain and large mobility deficits [1]. Obtaining pain relief and improving shoulder function are of significant concern to people with FS. Unfortunately, a definitive treatment for this condition does not currently exist and there is little consensus as to what constitutes optimal evidence-based treatment [2]. Despite some physical therapy interventions, such as joint mobilization and exercise, having shown therapeutic benefit [3–5], there is little evidence to suggest that the disease prognosis is affected [6]. Other interventions, such as guided intra-articular corticosteroid injections, appear to show more promising outcomes in the short-term than stand-alone physical therapy interventions [7]. Evidence also suggests the injection benefit being enhanced both in the short term and medium term when combined with physical therapy [8]. The current state of evidence for the various physical therapy treatments suggest that further and alternative approaches for managing FS might be investigated [6].

There is preliminary evidence from two systematic reviews showing that central pain processing mechanisms can contribute to the pain experience in a subgroup of patients with shoulder pain of different etiologies, including those with chronic subacromial impingement syndrome and post-stroke shoulder pain [9, 10]. Similarly, it could be argued that continuous nociceptive barrage, as in the early stages of FS, could lead to peripheral and subsequently long-lasting central sensitization. However, up to now the involvement of central mechanisms in FS remains speculative [6]. Interventions, such as pain neuroscience education and Graded Motor Imagery (GMI), which are thought to target the CNS, have been developed and tested in people with chronic musculoskeletal disorders with some promising results [11–15]. To our knowledge, only two case-series studies have used a CNS-focused treatment program in people with shoulder pain [16, 17]. In one study, a brief mirror therapy intervention resulted in statistically significant improvements in pain, pain catastrophization, fear avoidance and shoulder flexion active range of motion (ROM) in patients presenting with shoulder pain and limited active motion [16]. However, only 8.7% of the studied sample was diagnosed with FS and immediate post-intervention effects were solely assessed. In a second case series, Louw et al. showed that a sensory discrimination task applied to 55 patients with shoulder pain and limited ROM (including FS) resulted in an immediate increase of shoulder ROM (*p* = 0.001) with 25 patients (40%) meeting or exceeding minimal detectable change, but the study failed to report on the specific number of patients with FS [17]. Despite the positive effects shown in these two case series, the potential benefits of adding other approaches addressing the CNS (e.g., sensory discrimination training) remains largely unknown. Hence, further investigation of these preliminary findings in adequately powered randomized controlled trials together with exploration of the longer-term effects of centrally focused interventions for people with FS, is needed.

The aim of this study is to compare the effectiveness of a CNS-directed treatment program versus a standard medical and physical therapy care program on outcomes in participants with FS.

## Methods

### Design

This is a two-group, randomized clinical trial with blinding of participants and assessors.

### Setting

Participants will be recruited via referrals from orthopedic surgeons and physical therapists, community-based advertisements, private care practices and hospitals in Valencia, Spain. Potential referrals will be informed of the trial and the referral process via formal meetings and trial information sheets. This study is reported in line with the Standard Protocol Items; Recommendations for Interventional Trials (SPIRIT) Statement [18] (Additional file 1).

### Participants

Participants will be screened to determine whether they meet the following inclusion and exclusion criteria:

#### Inclusion criteria

Primary or idiopathic FS, defined as FS not associated with a systemic condition or history of injury [19]; greater than 50% reduction in passive external rotation when compared to the uninvolved shoulder or less than 30° of external rotation [20]; range of motion loss of greater than 25% in at least two movement planes in comparison to the uninvolved shoulder [20]; pain and restricted movement present for at least 1 month reaching a plateau or worsening [20]; normal shoulder x-rays (with the exception of osteopenia of the humeral head and calcific tendinosis) [21].

#### Exclusion criteria

Locked dislocations, rheumatic disease, fractures or avascular necrosis on radiographs; surgery in the upper quadrant region < 12 months prior to the study; skin or medical conditions that prevent patients from receiving tactile stimuli on the shoulder; neurological or motor disorders including a diagnosis of dyslexia or difficulty performing a rapid naming task; visual and mental health conditions that preclude successful participation.

### Details of the interventions

Participants will be randomized to receive either a CNS-focused treatment program or.

standard medical and physical therapy care. Adherence to both interventions will be monitored using an individual treatment diary where the time of day and duration of each clinic and home session will be recorded [22]. Adverse events will be recorded through passive capture. Patients will be requested to not participate in other treatments for their shoulder during the 10-week study period and any change in medication type or dosage during the study period will be recorded.

Trial physical therapists performing both interventions will have worked in private or public practice for at least 2 years. The clinicians performing the CNS-focused treatment will be engaged in a 1-day training session led by the author (ELL) for specific training in delivery of the interverventions comprising the program. This training session will include group discussions and quarterly workshops to review specific cases in the context of the CNS-focused treatment program. In addition, these physical therapists will be provided with a treatment manual outlining the CNS-focused treatment protocol and the details of each intervention included in the protocol. In order to ensure a good level of proficiency with the treatment protocol, trial physical therapists will go through a theoretical test and a practical exam with questions and techniques included in the protocol. The interventions are described in detail according to Template for Intervention Description and Replication (TIDieR) Checklist recommendations [23].

### CNS-focused treatment program

Participants randomized to this treatment will receive a CNS-focused intervention consisting of a 10-session treatment program delivered as 60-min sessions, scheduled once a week, over a period of 10 weeks. All treatment sessions are one-on-one. In addition, participants will complete a home treatment program entailing 30 min of training, five times per week that finishes at session 10. The intervention includes discussion of the participant’s shoulder pain experience from a pain neuroscience perspective (e.g., pain neuroscience education) [24], graded sensory discrimination training and GMI training. These interventions are likely to overlap due to variable allocation of time to each of the treatments within the clinic and home treatment sessions.

Prior to training, participants will be given an explanation of the proposed treatment and the aim of the study. Patients will be shown a picture of the “brain map” (homunculus) and taught how the map becomes “less sharp” when people are in pain, since the affected shoulder is not being moved [16]. They will be told that when the map is sharpened, it may help to reduce not only their pain but also their mobility [16]. By using sensory discrimination training and GMI, the therapy aims to sharpen the map of the shoulder in the brain and thus improve pain and movement.

#### Graded sensory discrimination training

A graded sensory discrimination training program based on previous work by Wand et al. [13] will be implemented. In this model, participants undertake a training regimen that involves discrimination of stimulus type and location and graphesthesia training in five different stages, graded according to level of theoretical cortical engagement and complexity. Each stage is planned to last a minimum of 2 weeks (10 weeks in total), but can be extended by some days if participants appear not to have sufficiently mastered that stage.

For tactile discrimination training in the first stage (weeks 0–2), participants will be seated in a comfortable position with a mirror between their upper limbs. Evidence has shown that tactile acuity is enhanced with visualization of the reflected image of the unaffected limb (that is, patients look towards the stimulated body part and can see the skin of the opposite body part in the mirror) [25]. Therefore, during the first week of training at home and in the clinic, participants will be positioned so that they can see the reflection of their unaffected arm in a mirror while the affected arm is stimulated. The limbs will be positioned in such a way that the reflected image of the opposite arm is in line with the stimulated arm. Visual feedback will be withdrawn after the first week and will not be used again in any part of the sensory training program.

In this first stage, only localization of the stimulus will be trained. Participants will be shown a digital standard photograph of the shoulder on which nine numbered grids will be marked. The spacing of the grids will be based on the current normative data pertaining to two-point discrimination of the affected joint (e.g., (45.9 mm ± 18.4 mm) [26]. For the shoulder localization blocks, the superior border will be set as 1 cm proximal to the acromioclavicular joint and the lower border reaching the deltoid insertion. While the participant views the photograph and nine-block grids, they will be taught via tactile stimulus with the back of the blunt end of a pencil, where each block is in relation to their shoulder, thus familiarizing them with the nine-block grid [13, 27]. After the familiarization period, the therapist, using a random number sequence, will press lightly on a particular point with the blunt end of a pencil for about 2 s. Pressure will be kept to a minimum to avoid pain provocation. Participants will be instructed to refer to the picture and to indicate which grid has been stimulated. With a correct identification of the area, the therapist will proceed to the next block for identification. If the participants make an error, they will be told which grid (number) has in fact been stimulated, and then the actual position of the grid that they have incorrectly indicated will be stimulated. This in essence will help the participant to develop a greater ability to identify the stimulated grid. Three blocks of 60 stimuli with an interstimulus interval of 15 s and a 3-min rest period between blocks will be used during the treatment session.

At the first session, participants will be accompanied by someone who can assist them to undertake training at home. This assistant will be trained in the task and participants will be advised to undertake 15 min of training at home in addition to the clinic session. Participants will be given a photograph of a standard shoulder on which the stimulation points will be marked and several sets of 60 random number sequences to use for training at home. If at the end of the second week (first stage), for participants who have less than 80% accuracy with one test block of 60 stimuli, the training will be extended for an additional week.

In the next stage (weeks 2–4), participants will be asked to discern both the localization of the stimulus (i.e., the corresponding number on the photograph) and the size of the probe used (type of stimulus). The experimental setup will be similar to that used in the first stage, but this time a probe with a sharp end (pen cap) and a blunt end (cork) will be used. A random number table will be used to randomize both position and probe size. Participants initially will be shown a picture with nine numbered grids marked on the shoulder; the number of grids will be increased to 12 in the second week of this stage. Again, participants will be given feedback about each error they make. Three blocks of 60 stimuli with an interstimulus interval of 15 s and a 3-min rest period between blocks will be used during the treatment session.

Should participants be less than 80% accurate with one test block of 60 stimuli at the end of the second week of this stage, then the training will be extended for an additional week. For home training in this second stage, participants will be given a photograph of the shoulder with the stimulation points and a wine cork and a pen lid to use as stimulus type. They will be given five lists of random combinations of numbers (1–9 or 1–12) and stimuli (cork or pen lid), and will be advised to use a different list each day. Participants will be advised to undertake 15 min of training at home in addition to the clinic session.

The next three stages (weeks 4–10) will involve graphesthesia tasks of increasing difficulty. In this third stage, participants will have to simply recognize letters drawn on the shoulder. Several random sequences of 60 letters will be generated, and three lots of 60 letters will be used in each treatment session with a interstimulus interval of 15 s and a 3-min rest period between blocks. Initially, uppercase letters will be drawn on the shoulder by the therapist with his index finger. Participants will be asked to indicate the letter drawn; if they guessed incorrectly, they will be told the actual letter that has been drawn, and then the letter that they have incorrectly indicated will be re-drawn. Progression within this 2-week block will be undertaken by decreasing the size of the letters, altering the orientation of the letters, and altering the speed at which the letters are drawn. Again, this stage may be extended by 1 week if participants are less than 80% accurate with a test block at the end of 2 weeks. Participants will be advised to undertake 15 min of graphesthesia training at home by using several random sequences of letters.

The next 2-week stage (weeks 6–8) will involve the recognition of three-letter words drawn on the shoulder. The protocol and progression will be almost identical to those outlined for the single-letter task, including the criterion for advancement to the next stage. One additional progression in the last 2 weeks (weeks 8–10) will involve overlapping the letters of the word such that they are all drawn on the same part of the shoulder. Again, this stage can be extended for an additional week if participants were less than 80% accurate at the end of 2 weeks. Participants will be advised to undertake 15 min of graphesthesia training at home by using several random sequences of letters.

A full description of the graded sensory discrimination training program is provided in Table 1.

**Table 1:** Summary of progressions used for the graded sensory discrimination training program

Stage	Sensory discrimination training
1 (weeks 0–2)	*Localization training* Determine site of stimulus With visual feedback during first week Without visual feedback during second week
2 (weeks 2–4)	*Localization and stimulus type* Determine site of stimulus Determine size of probe Progress by adding points
3 (weeks 4–6)	*Graphesthesia training* Recognize letters Progress by size Progress by orientation Progress by speed of drawing
4 (weeks 6–8)	*Graphesthesia training* Recognize 3-letter words Progress by size Progress by orientation Progress by speed of drawing Progress by overlapping letters
5 (weeks 8–10)	*Graphesthesia training* Progress by size Progress by orientation Progress by speed of drawing Progress by overlapping numbers

#### Graded Motor Imagery (GMI) training

A graded motor cortical retraining program based on previous work by Wand et al. [13] and published guidelines [28] will be implemented.

The initial stage (weeks 1–2) of the GMI will involve laterality recognition training (Implicit Motor Imagery). An online computer program (Recognise Online, NOI Group, Adelaide, SA, Australia) will be used to present participants with a random selection of photographs of either their left or right shoulders [28]. The photographs will be presented in a variety of positions and orientations. Participants will respond by pressing one of two keys to indicate whether a picture shows the left or right shoulder, a process that require them to mentally rotate their own body part to match the position shown in the picture and, thereby, to engage motor cortical areas corresponding to that body part. An important aspect of the test is that it is performed unconsciously (relatively) so it should be done as quickly as possible, almost as though the patient was guessing [28]. The photographs will be presented in groups of 30 for a duration of 5 s for each photograph, and progression will involve reducing the time for which the photographs are presented and changing the background of the photographs. During an initial familiarization session conducted during the first formal treatment, three lots of 30 photographs will be presented with a 1-min rest period between lots. Participants will be asked to practice this task at home for 15 min each day.

The next stage (weeks 3–4) will involve imagined movements (Explicit Motor Imagery). Two videos, each lasting approximately 7 min will be made of a person slowly performing a variety of shoulder movements from simple, low-load movements to more complex, behaviourally relevant movements. During the first week of this stage (week 3), the video will show small-range shoulder movements (e.g., unilateral shoulder flexion, extension, abduction, shoulder external and internal rotation in 0° of abduction). In the second week of this stage (week 4), the video will show a person performing the same movements as before but in full-range and more challenging and functional tasks (e.g., hand behind back, hand to curl hair). Participants will be in sitting in a relaxed position for imaging movements. They will be instructed to watch the videos and then close their eyes and to imagine themselves performing the same movements in a smooth and pain-free manner as if it was real in all its aspects, including the timing taken to move. Participants will be advised not to imagine watching themselves performing the movement but to imagine actually performing the movement in the first person. They will execute two series of 20 repetitions for every imagined movement in each session. Additionally, participants will be asked at home to watch the videos twice and to practice for a total of 15 min each day.

The next stage (weeks 5–6) will involve isometric contraction of the rotator cuff and scapulo-thoracic muscles using dynamic glenohumeral and scapulo-thoracic neuromuscular control exercises. It is believed that the activation of these muscles will serve as an ideal bridge between imagined movements and actual shoulder movements used in the next stage using mirror therapy (because there would not be shoulder movement, thus minimizing the potential for sensorimotor incongruence) and that the activation of these muscles might sharpen the cortical representation of the shoulder [13]. During the first week (week 5), participants will receive instruction on dynamic glenohumeral neuromuscular control exercises aiming to contract the rotator cuff muscles [29] and scapulo-thoracic muscles [30] in isolation. They will perform neuromuscular control exercises for three sets of 10-s repetitions with a 2-min rest period between sets. During the second week of this stage (week 6), the progression will involve maintenance of the local muscle contraction while participants move their shoulder in a pain-free manner in different directions. Exercise dose will be the same as during week 5. Participants will be asked to practice at home these tasks for a total of 15 min each day.

The next 4-week stage (weeks 7–10) will involve the use of mirror therapy with different progressions. Participants will be seated in a comfortable chair, towards the edge of the chair seat allowing for movement, but also providing some trunk support. The proposed mirror therapy will be demonstrated and explained to the subjects by the physiotherapist. Next, a standing mirror on wheels will be placed in front of the participant with the reflective side facing the uninvolved side. The affected arm will be placed behind the mirror. The participant will be asked to lean forward slightly, allowing them to view the complete uninvolved arm in the mirror. Mirror exercises will begin with simply watching the reflection of the unaffected arm in the mirror and then progressed from static to active and functional movements. When possible, gentle and synchronous movements of the affected arm will be encouraged behind the mirror. Two series of 12–15 min will be performed in each session, with 2 min between series to allow for resting and relaxing the arm. Additionally, participants will be asked to practice this task at home for 15 min each day with a mirror provided by researchers conducting the study.

Participants will be encouraged to move slowly and easily, breathing comfortably and focusing on the movement of the uninvolved arm. The intervention will allow subjects to move the uninvolved arm giving the “illusion” that their involved arm is moving through the full active ROM. Participants will be advised to stop if they have an increase in pain either during or directly after mirror therapy.

A full description of the GMI training program is provided in Table 2.

**Table 2:** Summary of progressions used for the Graded Motor Imagery (GMI) training program

Stage	GMI training
1 (weeks 0–2)	*Laterality recognition* Using Recognise software Determine whether left or right side of shoulder Progress by time for which image was presented
2 (weeks 2–4)	*Imagined movements* Using video of model performing movements Small-range movements during first week Full-range movements during second week
3 (weeks 4–6)	*Isometric local muscle recruitment* Rotator cuff muscles Scapular muscles Add pain-free movement to local contraction
4 (weeks 6–8)	*Mirror therapy* Keep the affected arm still in a comfortable position/keep the unaffected arm still in the same position and just observe the reflection Keep the affected arm still in a comfortable position/move the unaffected arm through its full-range of movement (ROM) in different directions
5 (weeks 8–10)	*Mirror therapy* Move the affected arm towards the limit of pain in the restricted/painful direction(s) of movement and keep that position/move the unaffected arm through its full ROM in the painful/limited directions Move the affected arm towards the limit of pain in the restricted/painful direction(s) of movement/copy with the unaffected arm through a full ROM (synchronous movements)

Should sustained symptom exacerbation occur in any of the stages, the appropiate parameters will be reviewed and possibly reduced.

### Standard medical and physical therapy care program

Participants randomized to standard medical and physical therapy care will receive a 10-session treatment program of the same duration as the CNS-focused treatment. This standard treatment will include one corticosteroid infiltration provided in the early acute stage followed by a multimodal physical therapy program including analgesic modalities (e.g., TENS, cryotherapy) and exercise and manual therapy techniques addressing the specific mobility deficits of each patient [31]. Physical therapists will be instructed not to include interventions that were similar to those used in the group receiving the CNS-focused protocol (e.g., using mirrors or imagined movements) and to include a home program that involves a training load comparable to that in the other group.

### Primary and secondary outcome measures and assessment points

The primary outcome measured is self-reported shoulder pain-related disability as measured on the Shoulder Pain And Disability Index (SPADI) questionnaire. The Spanish version of the SPADI has high internal consistency (Cronbach α: 0.916) and excellent test-retest reliability (ICC 0.91) [32]. Secondary outcomes are as follows:

1. The Numeric Pain Rating Scale (NPRS), a valid and reliable measure of shoulder pain [33]
2. Goniometric assessment of active shoulder ROM which is valid and reliable [34, 35]
3. Two-point discrimination threshold measured at one standardize site on the affected shoulder (5 cm distal to the lateral border of the acromion) [36], following an established protocol [37]
4. Laterality judgement accuracy using the NOI Recognize online program (www.noigroup.com) and following an established protocol [38]
5. The Spanish version of the Tampa Scale of Kinesophobia, a valid and reliable measure of fear of movement [39]
6. The Patient Specific Functional Scale, a reliable, valid and responsive instrument that can be used in patients with a primary shoulder complaint [40]

Assessment will occur at baseline, at the end of the treatment program (week 10), and at 3 and 6 months’ follow-up. At baseline, a clinical assessment of symptom distribution, history of the present and previous shoulder complaints, red flag screening, medical history and general health status will also be performed.

### Recruitment procedures

Participants will be recruited from different outpatient private clinics and rehabilitation services of different hospitals of the region of Valencia (Spain). In addition, posters will be distributed in the community and advertisements in social media will be placed to increase the potential number of participants in the study. Physical therapists and primary care practitioners will be contacted and invited to recruit participants after providing them with brief information about the study. Involved practitioners will identifiy potentially suitable patients and, after providing them with information about the study, will invite them to contact the research team. Upon contact by potential participants, a researcher will explain the study and assess them for study eligilibily via telephone. If the potential participant remains interested in participating in the study, they will be invited to a baseline session. During that session, one researcher will provide to the patient an information leaflet, confirm eligibility, and obtain a signed consent form. Baseline outcome data will be collected during this session, following which the participant will be randomized.

Adherence to treatment will be enhanced by careful explanation of the time demands of participation and regular contact by a researcher who will send repeated reminders to participants by email and make telephone calls to ensure adherence to the time schedule including follow-up sessions.

**Fig. 1:**
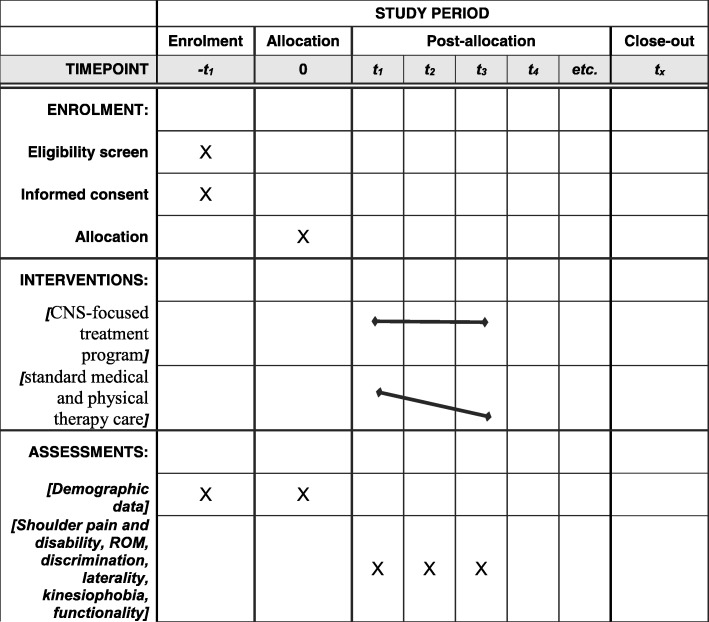
Schedule of enrollment, interventions and assessments

The schedule of the enrollment, interventions and assessments is shown in Fig. 1.

### Randomization procedures

Randomization will be conducted using computer-generated random numbers (Epidat® version 3.1). The allocation sequence will be prepared by a researcher with no involvement in the study by using a blocked randomization model. Allocation concealment will be ensured using 34 sequentially numbered opaque and sealed envelopes. After performing the baseline assessments the treating clinician will open the envelope and reveal each participant’s group allocation.

### Blinding

Participants will be blinded to both study hypothesis and group allocation. It will not be possible to blind the treating physical therapists who are responsable of performing the interventions. All the assessments will be conducted by researchers who will be blinded to group allocation. Statitistical analysis will be performed by a statistician blinded to the study aims.

### Statistical analysis including sample size calculation

#### Sample size calculations

The sample size will be calculated using G*Power 3.0.18 Software based on the SPADI as the primary outcome measure. To our knowledge, there are no studies investigating the effects of GMI or graded sensory discrimination training on FS. Based on similar studies applying physiotherapy on FS (SPADI mean of 66 points; standard deviation (SD) = 16) [8], and the minimal detectable change attained in the study by Tveita et al. (17 points) [41], to detect a 17-point (SD = 16) between-group difference, with 80% power and an alpha level of 0.05, a total sample size of 30 patients is estimated (15 per group). An allowance will be made for a 15% dropout rate, increasing the sample size to 34 patients (17 per group). However, since this calculation is not based in the use of GMI, to assure an adequate sample size, we will carry out a pilot study with 20 participants (10 per group) to test these assumptions. Mean differences and standard deviations from the inter-group comparison on the primary outcome (SPADI) will then be used to recalculate the sample size, if necessary.

#### Statistical analysis

Data will be analyzed using the statistical package SPSS 21.00 for Windows. Statistical significance will be set at *p* < 0.05. Prior to statistical comparisons, all data will be tested for normal distribution. Then, a descriptive analysis of the data will be obtained for the dependent variables in the different assessment times. Subsequently, homogeneity of the two intervention groups will be studied. To confirm if there are differences in each group (intra-group comparisons), considering each group in isolation, between the four assessments in each of the variables (baseline, post treatment, 3-month follow-up, 6-month follow-up), repeated measures analysis of variance ANOVA will be used. To calculate inter-group differences between baseline and follow-ups, a four-way repeated-measures ANOVA will be conducted, with the scores of every primary and secondary outcome as dependent factors, with four levels corresponding to every time of assessment (t1, t2, t3 and t4), and the two intervention groups (CNS-focused treatment vs standard care treatment) as independent factors. Between- and within-group effect sizes for all quantitative variables will be measured with the Cohen *d* coefficient. An effect size greater than 0.8 will be considered large, around 0.5 moderate, and less than 0.2 small [42]. In cases of missing data, an intention-to-treat analysis will be performed. Double data entry will be carried out in order to promote data quality.

### Data management

Data from the study will be only accessible to the research team and will be stored on password-protected computers at the University of Valencia. Paper-form data will be stored in locked cabinets located at the Department of Physiotherapy of that same university. In order to preserve data confidentiality study participants will be assigned an identification number which will be kept for the duration of the study. A list of participant identification numbers will be created and separated from the de-identified data. Statistical analyses will be performed keeping participant anonymity by using patient identification numbers and the statistician will be blinded to group allocation. Confidentiality will also be preserved when disseminating results by using group data.

### Significance and implications for practice

Preliminary data suggest that treatments that target CNS function are a promising approach to the treatment of people with shoulder pain including patients with FS. In the context of modest effects from most available physical therapy treatments for FS, this CNS-focused approach may lead to improved clinical outcomes. The trial should determine if the CNS-directed program is more effective than traditional interventions at reducing pain intensity and improving function in a FS cohort and will follow up participants for 6 months, providing important information on the persistence of any treatment effects. The inclusion of variables related to functional reorganization of the brain, such as the two-point discrimination threshold and laterality judgement accuracy, will also allow for the first time to explore responsiveness to change of these tests after treatment in a population with shoulder pain. In addition, this study provide a good oportunity to explore the relationship between shoulder pain, cortical changes and clinical markers in people with FS. Finally, the flexible structure of the interventions comprising the CNS-focused approach closely reflects the real-world clinical practice.

CNS-directed interventions constitute a completely new treatment paradigm for the management of shoulder pain and, in particular, people with FS. Feelings of stiffness in the back have been recently demonstrated to be a multisensory perceptual inference consistent with protection rather than reflecting biomechanical properties of the back [43]. Stiffness is a main characteristic in people with FS and the prevailing view is that it is related to a capsular fibrosis despite the cause being still unknown [44]. The positive effects in ROM observed in preliminary research conducted in people with FS after brief interventions targeting the CNS challenge the prevailing view that stiffness in FS is an isomorphic marker of the biomechanical characteristics of the shoulder. The results of this study should have the potential to address this issue and change the current physiotherapy management of FS.

### Anticipation dates of trial commencement and completion

Commencement March 2018. Completion September 2020.

### Ethics and dissemination

The trial has been registerd at Clinicaltrials.gov with the identifier: NCT03320200. The results of the study will be disseminated at several research conferences and as published articles in peer-reviewed journals. The full protocol, participant-level dataset, and statistical code will be available when this study will be finished.

## Additional file

Additional file 1: Standard Protocol Items; Recommendations for Interventional Trials (SPIRIT) 2013 Checklist: recommended items to address in a clinical trial protocol and related documents. (DOC 125 kb)

## Data Availability

Not applicable.
